# Understanding the Experience of Long COVID Symptoms in Hospitalized and Non-Hospitalized Individuals: A Random, Cross-Sectional Survey Study

**DOI:** 10.3390/healthcare11091309

**Published:** 2023-05-03

**Authors:** Jacqueline A. Krysa, Mikayla Buell, Kiran Pohar Manhas, Katharina Kovacs Burns, Maria J. Santana, Sidney Horlick, Kristine Russell, Elizabeth Papathanassoglou, Chester Ho

**Affiliations:** 1 Neurosciences, Rehabilitation and Vision, Strategic Clinical Network, Alberta Health Services, Edmonton, AB T5J 3E4, Canada; 2 Division of Physical Medicine and Rehabilitation, University of Alberta, Edmonton, AB T6G 2E1, Canada; 3 Community Health Sciences, Cumming School of Medicine, University of Calgary, Calgary, AB T2N 1N4, Canada; 4 School of Public Health, University of Alberta, Edmonton, AB T6G 1C9, Canada; 5 Department of Clinical Quality Metrics, Alberta Health Services, Edmonton, AB T5J 3E4, Canada; 6 Faculty of Nursing, University of Alberta, Edmonton AB T6G 1C9, Canada

**Keywords:** long COVID, hospitalization, patient experience, COVID-19 recovery, COVID-19 severity and impact

## Abstract

The relationship between initial COVID-19 infection and the development of long COVID remains unclear. The purpose of this study was to compare the experience of long COVID in previously hospitalized and non-hospitalized adults in a community-based, cross-sectional telephone survey. Participants included persons with positive COVID-19 test results between 21 March 2021 and 21 October 2021 in Alberta, Canada. The survey included 330 respondents (29.1% response rate), which included 165 previously hospitalized and 165 non-hospitalized individuals. Significantly more previously hospitalized respondents self-reported long COVID symptoms (81 (49.1%)) compared to non-hospitalized respondents (42 (25.5%), *p* < 0.0001). Most respondents in both groups experienced these symptoms for more than 6 months (hospitalized: 66 (81.5%); non-hospitalized: 25 (59.5), *p* = 0.06). Hospitalized respondents with long COVID symptoms reported greater limitations on everyday activities from their symptoms compared to non-hospitalized respondents (*p* < 0.0001) and tended to experience a greater impact on returning to work (unable to return to work—hospitalized: 20 (19.1%); non-hospitalized: 6 (4.5%), *p* < 0.0001). No significant differences in self-reported long COVID symptoms were found between male and female respondents in both groups (*p* > 0.05). This study provides novel data to further support that individuals who were hospitalized for COVID-19 appear more likely to experience long COVID symptoms.

## 1. Introduction

In Canada, 4.42 million individuals have recovered from COVID-19, of which over 100,000 were hospitalized [1]. The World Health Organization defines post-COVID conditions as any new, recurring, or lingering symptoms that persist for at least 12 weeks following acute COVID-19 infection and cannot be explained by an alternative diagnosis [2]. Long COVID is a more general term used to describe new or ongoing signs and symptoms that develop after recovery from acute COVID-19 and includes ongoing symptomatic COVID-19 (4–12 weeks since initial recovery) and post-COVID conditions (12 weeks or more since initial recovery) [3]. Current estimates of long COVID prevalence vary regionally and between COVID-19 variants [4,5]. The Canadian COVID-19 Antibody and Health Survey (CCAHS-2) described the experience of long-term symptoms after a COVID-19 infection between January 2020 and August 2022 [6]. Prior to December 2021 (pre-Omicron variant), one in four persons with confirmed or suspected COVID-19 infection reported long COVID symptoms [6]. Conversely, of those that experienced COVID-19 after December 2021, about one in ten persons reported long COVID symptoms [6]. This highlights the changing severity and experience of long COVID over time.

Long COVID includes common, diverse, and varying symptomology that affect multiple organ systems, as well as functional, cognitive, and mental health outcomes [7,8,9,10,11,12]. Common long COVID symptoms include anosmia, anxiety, cognitive problems, exercise intolerance, fatigue, headaches, impaired sleep, and shortness of breath [2,12,13,14,15]. These lasting health consequences, manifesting as chronic symptoms, have been found to significantly impact physical and cognitive function, participation in daily activities, and overall quality of life [16]. The underlying pathophysiology and long-term impact of these symptoms remain largely unknown [4,5].

Considering the recency and burden of long COVID on persons recovering from COVID-19, there is increasing interest in understanding the relationship between initial COVID-19 recovery and the development of long COVID [17]. Although long COVID is frequently observed in those that experienced milder forms of acute COVID, recent evidence indicates some association between hospitalization for COVID-19 and an increased likelihood of developing long COVID [17,18,19,20,21,22,23]. A retrospective cohort study of 133 inpatients that tested positive for COVID-19 found that 64.7% self-reported long COVID symptoms four months after the index date (the date on which hospitalization for COVID-19 occurred) [24]. Conversely, individuals who were not hospitalized for COVID-19 appeared less likely to develop long COVID [25]. One study reviewed over 400,000 recorded COVID-19 cases in non-hospitalized adults between January 2020 and April 2021 and found that 5.4% of patients reported at least one long COVID symptom 12 weeks after the index date [25]. This evidence suggests a role for early hospitalization in the development of long COVID and has Implications for those more at risk and requiring hospitalization, including older age groups and those with underlying conditions [24,26].

Evidence on the impact of previous hospitalization for acute COVID-19 on the development of long COVID is emerging [27,28,29]. A 2022 systematic review and meta-analysis found that independent of hospitalization status, 45% of COVID-19 survivors experienced a diversity of unresolved symptoms after 4 months [27]. Moreover, a 2022 prospective cohort study reported that long COVID symptoms were more common in hospitalized patients compared to outpatients after 6 months (52.3% vs. 38.2%, respectively) [28]. Due to the limited availability of validated tools and lack of a unified definition of long COVID during the pandemic, most studies rely on the self-reporting of symptoms to understand the impact of long COVID [30]. Given the current limitations to identifying long COVID with lower self-selection bias and the challenges of monitoring recovery outcomes following COVID-19, more evidence is required to determine the relationship between previous hospitalization and the development and severity of long COVID. To better understand the relationship between previous hospitalization and long COVID, this study aimed to compare the patient experience of long COVID following a positive COVID-19 test in a random sample of previously hospitalized and non-hospitalized adults.

## 2. Materials and Methods

### 2.1. Study Design

In this cross-sectional, provincial, observational study, a telephone survey was administered to a random sample of persons recovering from COVID-19 to understand their experience with long COVID and navigating health services. This study was approved by the University of Alberta Research Ethics Board (Pro00113182) and complied with all relevant guidelines and regulations. All respondents consented to participate in this study. Reporting was guided by the Checklist for Reporting of Survey Studies (CROSS) (Supplemental Table S1) [31].

### 2.2. Study Population

Persons recovering from acute COVID-19 infection in Alberta, Canada, were recruited. Inclusion criteria were individuals aged >18 years with a laboratory-confirmed COVID-19 polymerase chain reaction (PCR) test between 21 March 2021 and 21 October 2021 and able to read and understand English (with or without the assistance of an available family member/friend). There were no explicit exclusion criteria for study respondents.

### 2.3. Sampling

This study aimed to recruit 300 respondents. This number was estimated to ensure representation within each stratum, the feasibility of recruitment, and to account for limited numbers of previously hospitalized, COVID-19 positive individuals. This estimated target was also selected to reduce the burden of survey fatigue and to account for the sensitivity and emotional context of COVID-19, as these factors can impact the survey response rate [29]. Professional and experienced telephone surveyors developed a proportional stratified random sampling frame based on pre-specified inclusion criteria and applied this to existing administrative databases for hospitalized and non-hospitalized individuals with a positive COVID-19 test. The sampling frame was based on hospitalization status during the study window (50% hospitalized and 50% non-hospitalized) and geographical location (60% metropolitan-urban residence and 40% regional-urban/rural residence). Alberta is a province in western Canada with five geographical zones with an overall population of 4.54 million [32]. The 2021 Census profile of Alberta reported that approximately 50% of residents were female, and the most commonly reported ethnic backgrounds included English (18.0%), German (15.0%), Scottish (14.8%), and Irish (13.2%) [33]. In Alberta, two zones, Calgary and Edmonton, represent metropolitan-urban regions, with densely populated areas exceeding 100,000 individuals. The other three zones, North, Central, and South, represent regional-urban/rural areas, with generally between 10,000 and 100,000 individuals in densely populated regions [33].

Telephone surveyors randomly selected and called eligible individuals from relevant administrative databases and would call back those that agreed or continued to call those that did not respond to the initial call for up to three-call backs, as needed. This process ensured that all randomly selected individuals had the opportunity to respond to or decline the survey. Telephone surveyors would continue to draw random samples until targeted numbers of cases and strata were achieved. Recruitment continued past the target of 300 respondents to further minimize response and non-response bias and ensure saturation of data responses.

### 2.4. Survey Content Development

**Figure 1:**
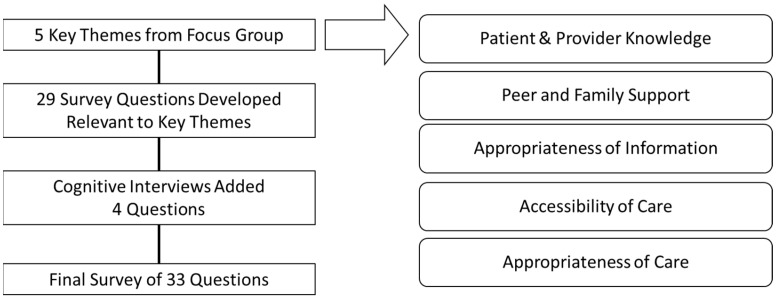
Item Selection in the Development of a Long COVID Patient Experience Survey.

The survey was co-designed with input from provincial stakeholders, including patient and family advisors with lived experience of long COVID, clinicians, and administrative, operational, and health system leadership. Figure 1 summarizes the process involved in the survey design. To inform the co-design process, a one-time virtual focus group was held with provincial stakeholders and patient and family advisors to determine priority concepts regarding the patient experience of navigating long COVID care to inform health service planning. Five core concepts emerged from the focus group: patient and provider knowledge, appropriateness of the information, peer and family support, accessibility of care, and appropriateness of care. These core concepts informed the development of the draft survey. Five cognitive interviews were conducted with persons with lived experience of long COVID to reduce the likelihood of item non-response error and measurement error [34,35]. Verbal probing was used throughout the cognitive interviews to explore how respondents understood and answered survey questions. The probes were used to assess concern about the content of the survey questions, survey construction, overall comprehension, and possible reactions from respondents for each question. Textual analysis of interview notes allowed for comparison of responses across respondents for each item. These interviews led to changes in survey content, formatting, and layout to determine the final version of the survey. An additional literacy/language correction to grade 5/6 level was completed. The final survey and results of the survey were shared with participating patient and family advisors by email and presentation. The final survey was designed for online or phone delivery, consisting of 33 questions (including 6 demographic questions) (see Supplemental File S2). Survey items included closed, multiple-response, and open-ended questions. Survey questions probed to better understand the experience or lack of experience of prolonged symptoms after COVID-19 recovery, as well as the impact of long COVID symptoms on daily activities and return to work. In order to identify those with long COVID symptoms, respondents were asked whether they were experiencing ‘any new or lasting/ongoing symptoms after recovery from COVID-19’. This wording was recommended to avoid confusion about the definition of long COVID by respondents. Prior to the start of the survey, telephone surveyors overviewed the definition of long COVID to ensure participants could differentiate between the experience of COVID-19 recovery and long COVID. Additional questions (not discussed in the present manuscript) included general and specific experiences regarding health system navigation, as well as open-ended questions on perceived challenges, positive experiences, and improvement suggestions.

### 2.5. Recruitment and Data Collection

Eligible hospitalized and non-hospitalized COVID-19 respondents were identified through Alberta Health Services’ administrative COVID-19 databases between April and June 2022. Potential respondents within the hospitalized and non-hospitalized databases were randomized and recruited by health system-employed, professionally trained telephone surveyors [36]. Surveyors provided a brief study introduction and outlined participation requirements over the telephone. Those that agreed to participate provided verbal consent. Survey questions were read to the study respondents by the telephone surveyors, who then recorded their responses. Survey responses were recorded and stored in VOXCO, a secured and private survey software (VOXCO Survey Software, Montréal, QC, Canada, 2022). Only select telephone surveyors had access to VOXCO to prevent unauthorized access. Recruitment continued until stratification and a minimum sample size of at least 150 hospitalized and 150 non-hospitalized individuals was reached.

### 2.6. Data Analysis

Survey data were de-identified and cleaned for analysis by telephone surveyors before analysis by the research team. SPSS (IBM SPSS Statistics 25, New York, NY, United States) was used to analyze data. Descriptive statistics were used to describe and summarize the results. Comparisons of proportions between groups (including hospitalization status, gender, and geographical location) were examined using Pearson’s Chi-square test of independence. Independent comparisons were conducted between self-identified ethnicity as respondents were invited to select multiple options. Missing data (either due to omitted answers or ‘not applicable’ answers) is reported for all variables and was adjusted for in the analysis. Statistical significance was set at *p* < 0.05.

## 3. Results

### 3.1. Response Rate and Respondent Demographics

**Figure 2:**
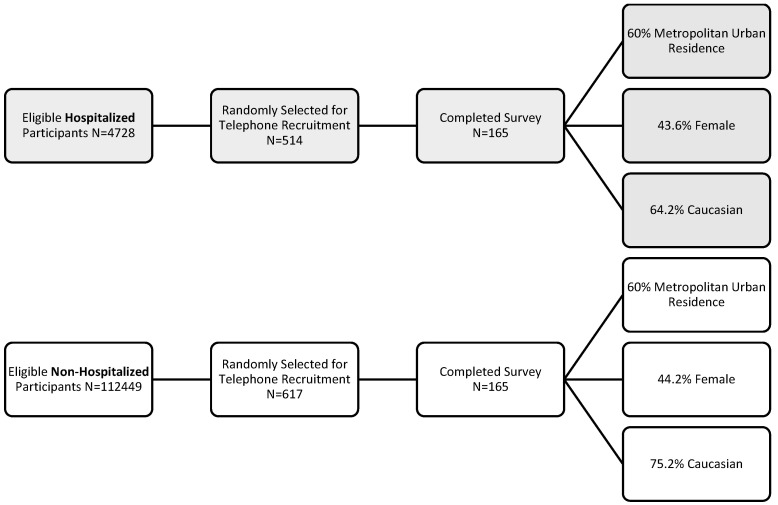
Survey Response Rate.

A total of 1131 persons were invited to participate in the survey. Telephone surveyors contacted 514 previously hospitalized individuals that were randomly selected from a larger pool of 4728 eligible participants. Of these, 222 were disqualified, 112 refused to participate, 15 had indeterminant responses, and 165 completed the survey (59.6% response rate). Out of 112,449 eligible non-hospitalized participants, 617 were randomly selected. Of these, 305 were disqualified, 6 had indeterminant responses, 141 refused to participate, and 165 completed the telephone survey (53.92%). In total, the survey had 330 respondents (Figure 2). The telephone survey took between 7 and 64 min (median 10 min) to complete. Table 1 reports the demographics of the 330 survey respondents. Per pre-defined sampling strata, 50% of the COVID-19 positive respondents experienced hospitalization due to COVID-19 and 60% of respondents in both groups lived in a metropolitan-urban residence. Hospitalized respondents reported significantly different age groups than non-hospitalized respondents, with more respondents being in older age groups (*p* < 0.0001). 72 (43.6%) previously hospitalized respondents and 73 (44.2%) non-hospitalized) identified as female (*p* = 0.99). Significantly more non-hospitalized respondents self-identified as Caucasian (106 (63.5%) compared to previously hospitalized respondents (124 (75.9%), *p* = 0.03). Employment status prior to a positive diagnosis of COVID-19 was significantly different between groups (*p* < 0.0001), with more than 74 (44.8%) of the previously hospitalized and 100 (60.6%) of the non-hospitalized respondents self-reported full-time employment status.

### 3.2. Self-Reported Post-COVID Symptoms by Hospitalization Status

Almost half of the previously hospitalized respondents self-reported long COVID symptoms following a positive diagnosis of COVID-19 (81 (49.1%)). This was significantly greater compared to self-reported symptoms by non-hospitalized respondents (42 (25.5%), *p* < 0.0001) (Table 2). Most respondents in both groups self-reported long COVID symptoms lasting for longer than 6 months (hospitalized 66 (81.5%); non-hospitalized 25 (59.5%), *p* = 0.06)). Previously hospitalized respondents reported significant impairment from their long COVID symptoms on their everyday activities (24 (29.6%)) compared to non-hospitalized respondents (1 (2.4%), *p* < 0.0001). Of the individuals that reported full-time working status prior to COVID-19, significantly more non-hospitalized respondents (115 (85.8%)) were able to return to this work after recovery compared to previously hospitalized respondents (65 (61.9%, *p* < 0.0001) (Table 2).

### 3.3. Self-Reported Long COVID by Gender

Table 3 reports a comparison of self-reported long COVID symptoms by gender and hospitalization status. No significant differences were observed in self-reported long COVID symptoms between male and female respondents in hospitalized (males 42 (45.6%); females: 39 (52.2%), *p* = 0.34)) and non-hospitalized groups (males 18 (19.7%); females 23 (31.0%), *p* = 0.10). Significantly fewer female respondents were able to return to their previous level of employment since their experience of long COVID (males 43 (46.7%), females 21 (29.1%)). No significant differences in returning to work were observed between males and females in the non-hospitalized groups (males 66 (72.5%), females 48 (65.7%), *p* = 0.91). Comparable duration of symptoms and impact of symptoms on daily activities were reported between genders in both groups (*p* > 0.05).

### 3.4. Comparison of Self-Reported Long COVID by Geographical Region

Table 4 reports on respondents from metropolitan-urban residences compared to those from regional-urban/rural residences that were or were not hospitalized for COVID-19. Comparable self-reported long COVID symptoms and the impact of symptoms on daily activities were reported between metropolitan-urban and regional-urban respondents amongst previously hospitalized and non-hospitalized groups (*p* > 0.05).

## 4. Discussion

The COVID-19 recovery trajectory and experience of long COVID symptoms remain unclear [37]. In this present study, we investigated the experience of COVID-19 recovery in a random sample of individuals that tested positive for COVID-19 between the 21 March 2021 and the 21 October 2021. Our results found that individuals previously hospitalized for COVID-19 were more likely to self-report long COVID symptoms and experienced a greater duration and severity of symptoms compared to those that had not been hospitalized. Due to the recency and emerging evidence on underlying risks and outcomes of long COVID, this study contributes to rigorous, generalizable knowledge to advance our understanding of long COVID in the general population.

Our findings align with the recent Canadian CCAHS-2 survey, which found that respondents with more mild, initial COVID-19 symptoms reported fewer longer-term symptoms than those that reported moderate symptoms (6.3% versus 15%, respectively) [6]. More severe acute COVID-19 infection has been identified as a significant risk factor for developing long COVID [6,23,38,39]. A 2021 systematic review of ten international cohort studies revealed that individuals with milder acute COVID-19 experienced a faster estimated recovery (median duration of symptoms: 3.99 months (interquartile range (IQR) 3.84–4.20)) compared to those admitted for hospitalization for acute infection (median duration of symptoms: 8.84 months (IQR 8.10–9.78)) [38]. Additional findings from this systematic review found that 15.1% (IQR 10.3–21.1) of respondents continue to experience long COVID symptoms after one year [38]. The present study adds and strengthens these findings by directly comparing the experience of long COVID in previously hospitalized and non-hospitalized respondents and further emphasizes the relationship between the severity of acute COVID-19 symptoms and the development of long COVID symptoms.

Further analysis of our survey findings revealed comparable self-reported long COVID, duration of symptoms, and impact of symptoms on daily activities between male and female respondents. This contrasts with recent findings from the Canadian CCAHS-2 survey, which demonstrated a higher percentage of women experiencing long COVID symptoms compared with men (18.0% versus 11.6%, respectively) [6]. A recent study explored risk factors associated with long COVID in over 480,000 COVID positive adults, finding that the female sex was a significant risk factor for developing long COVID (adjusted Hazard Ratio 1.52 95% CI 1.48–1.56) [25]. The lack of significant difference in long COVID symptoms between males and females in this study may be due to the approach for self-reporting long COVID symptoms.

Common long COVID symptoms, such as fatigue, dyspnea, muscle weakness, and mood disturbances, can significantly impact an individual’s ability to perform activities of daily living (ADLs) [40]. ADLs describe the collective, fundamental skills necessary to independently care for oneself (e.g., mobility, eating, and bathing) [41,42]. The Canadian CCAHS-2 survey revealed that 21.3% of adults with self-reported PCC symptoms experienced limits in their daily activities [6]. In a systematic review (n = 9 articles), ADL performance significantly declined following COVID-19 infection [40]. Similarly, 7.9% of respondents from a longitudinal prospective cohort of adults with laboratory-confirmed COVID-19 reported negative impacts to at least one ADL up to 9 months after their acute infection [43]. In this present study, a survey item modified from the post-COVID functional scale (PCFS) was used to assess the impact of long COVID symptoms on function and ADLs [44]. The PCFS is an ordinal tool designed to measure and track functional outcomes, focusing on changes in ADLs following COVID-19 [44]. PCFS scoring ranges from grade 0–4, where grade 0 reflects the absence of functional limitations and grade 4 reflects severe functional limitations requiring assistance with ADLs [44]. Our survey revealed that almost one-third of previously hospitalized respondents with self-reported PCC symptoms reported severe limitations to their everyday activities (comparable to PCFS grade 4), compared to very few (2%) of non-hospitalized respondents with self-reported long COVID. A cross-sectional study of over 100 patients recovering from COVID-19 identified that more than half (56.6%) of respondents reported no functional limitations (PCFS grade 0), while 43.4% of respondents indicated some degree of functional limitations (grade 1 to 4) [45]. By designing a survey item based on the PCFS, our survey was able to clarify that previous hospitalization was associated with greater severity of long COVID symptoms and impact on ADLs.

The persistence and impact of long COVID symptoms on ADLs have significant implications for returning to work [46]. Our survey results indicated that over 30% of previously hospitalized respondents and 10% of non-hospitalized respondents were unable to maintain their previous level of employment following recovery from COVID-19. Results from the Wuhan follow-up cohort study revealed that 12 months after hospitalization for COVID-19, 12% of respondents had not returned to work, and 24% of respondents were unable to return to their pre-COVID level of work [47]. Likewise, a patient-led survey of over 3700 individuals with suspected or confirmed COVID-19 reported that 45.2% had a reduced work schedule compared to pre-COVID, and 22.3% were not working at the time of the survey because of their current health condition [15]. Our study was able to report changes in employment status up to 13 months following initial infection and adds to the growing evidence of the impact of long COVID on productivity and the global workforce.

The strengths of this study include the process followed by professional, experienced telephone surveyors to support study recruitment. The random sampling approach resulted in respondent demographics that aligned closely with the population characteristics of the province of Alberta and, therefore, is a representative sample [29,30]. The survey was conducted between April 2022 and June 2022, allowing between 6 and13 months of follow-up, which allows for a better understanding of the longer-term COVID-19 recovery trajectory. Another strength of the survey was the co-design of a novel survey tool by a multidisciplinary group of stakeholders, including patient and family advisors, refined using cognitive interviews. This allowed for an investigation of experience regarding a novel condition based on the experiences and needs of professionals caring for and persons living with long COVID. The novelty of this tool is also a limitation, as this tool has not been validated to assess long COVID symptoms and, therefore, limits comparability between subjects or studies. Additional limitations of this study included an estimated sample size and self-reporting of long COVID symptoms, which may misrepresent the true prevalence of long COVID in this sample. This study also focused on individuals that experienced COVID-19 in Waves 3 and 4 of the pandemic in Canada (between March 2021 and October 2021). Therefore, the experiences of respondents may not reflect the experiences of those who had COVID-19 prior to March 2021 or after October 2021. Recent studies have indicated that the Omicron COVID-19 variant and vaccination may have contributed to a decrease in the likelihood of developing long COVID compared to earlier variants [6,48,49]. In the present study, we did not retrieve the vaccination status of our respondents, which limited inferences about the impact of vaccination status and COVID-19 recovery. Although we were able to stratify sampling by metropolitan-urban and regional-urban/rural areas of the province, we did not have adequate numbers of COVID positive individuals, either hospitalized or non-hospitalized, to directly compare rural and urban living status. Therefore, we were not able to accurately describe all the differences we observed between groups. The present survey was also not designed to include questions regarding socioeconomic status, anthropometrics, lifestyle habits (i.e., smoking, alcohol use), or pre-existing conditions. Additional risk factors identified for long COVID include belonging to an ethnic minority group, a gradient of decreasing age, socioeconomic deprivation, smoking status, high body mass index, and presence of comorbidities [25]. Respondents in the survey were English-speaking and primarily identified as Caucasian (64% hospitalized and 75% non-hospitalized), and, therefore, the results may not be generalizable to other ethnic/racial groups. Future studies should investigate the prevalence, incidence, and experience of long COVID in other diverse populations to better understand the types of recovery services required.

## 5. Conclusions

The underlying pathophysiology recovery trajectory of long COVID remains unclear [38]. This study provides novel insights into the recovery from COVID-19 and the experience of long COVID by directly comparing self-reported long COVID symptoms as well as the perceived impact of symptoms on everyday activities and returning to work in previously hospitalized and non-hospitalized individuals. It also supports that individuals at greater risk of hospitalization from COVID-19 may have an increased likelihood of developing long COVID and require additional follow-up care and supports after discharge. Collectively, these findings add to the growing evidence on the existence and impact of long COVID and have implications for the development of novel care pathways and post-hospitalization follow-up to support patient recovery, returning to work, and overall quality of life.

## Figures and Tables

**Table 1:** Survey Respondent Demographics by Hospitalization Status.

Item	Hospitalized (n = 165)	Non-Hospitalized (n = 165)	*p* Value χ^2^
Age Group (Years)			<0.0001
18–24	0 (0.0%)	16 (9.7%)	
25–40	23 (13.9%)	61 (37.0%)	
41–55	43 (26.1%)	53 (32.1%)	
56–65	45 (27.3%)	19 (11.5%)	
66–75	42 (25.5%)	9 (5.5%)	
>75	12 (7.3%)	6 (3.6%)	
Refused	-	1 (0.6%)	
Female Gender	72 (43.6%)	73 (44.2%)	0.99
Metropolitan-Urban Residence.	99 (60%)	99 (60%)	1.00
Racial/Ethnic Groups *			
Arab	5 (3.0%)	1 (0.6%)	0.10
Black	4 (2.4%)	5 (3.0%)	0.74
Caucasian	106 (63.5%)	124 (75.9%)	0.03
Chinese	2 (1.2%)	3 (1.8%)	0.65
Filipino	11 (6.6%)	8 (4.8%)	0.48
Indigenous	8 (4.8%)	6 (3.5%)	0.56
Latin American	4 (2.4%)	4 (2.4%)	1.00
South Asian	7 (4.2%)	9 (5.3%)	0.61
Southeast Asian	3 (1.8%)	2 (1.2%)	0.65
West Asian	1 (0.6%)	0 (0.0%)	0.32
Other	16 (9.6%)	8 (4.7%)	0.09
Hospital Length of Stay (Days (mean, SD))	9.4 (12.6)	N/A	N/A
What was your employment status before you were infected by COVID-19?			<0.0001
Full-Time	74 (44.8%)	100 (60.6%)	
Part-Time	7 (4.2%)	13 (7.9%)	
Casual	3 (1.8%)	4 (2.4%)	
Student	2 (1.2%)	7 (4.2%)	
Not Employed	16 (9.7%)	15 (9.1%)	
Retired	44 (26.7%)	16 (9.7%)	
Other	19 (11.5%)	10 (6.1%)	

* Participants were invited to select multiple options as appropriate to their racial/ethnic identity.

**Table 2:** Self-Reported Long COVID Symptoms by Hospitalization Status.

Item	Hospitalized (n = 165)	Non-Hospitalized (n = 165)	*p* Value χ^2^
Are you having new or lasting/ongoing symptoms (e.g., physical, cognitive, emotional, etc.) since you first tested positive for COVID-19?			<0.0001
Yes	81 (49.1%)	42 (25.5%)	
No	78 (47.3%)	118 (71.5%)	
Do Not Know	6 (3.6%)	5 (3.0%)	
How long have you been experiencing these symptoms?			0.06
<1 Month	4 (4.9%)	3 (7.1%)	
1–3 Months	2 (2.5%)	2 (4.8%)	
3–6 Months	8 (9.9%)	12 (28.6%)	
>6 Months	66 (81.5%)	25 (59.5%)	
Do Not Know	1 (0.6%)	-	
Since your experience of long-COVID, have you been able to return to your previous employment status?			<0.0001
Yes	65 (61.9%)	115 (85.8%)	
Partially	15 (14.3%)	6 (4.5%)	
No	20 (19.1%)	6 (4.5%)	
Other	3 (2.9%)	5 (3.7%)	
Please select the choice that best describes how your symptoms could have had an impact on your usual, everyday activities.			<0.0001
My Usual, Everyday Activities are Not Impacted by My Symptoms.	10 (12.3%)	17 (41.5%)	
I Can Perform Most of my Usual Activities.	8 (9.9%)	8 (19.5%)	
I Sometimes Need to Stop or Cut Down on My Usual Activities.	23 (12.3%)	12 (29.3%)	
I Often Need to Stop or Cut Down My Usual Activities.	10 (12.3%)	3 (7.3%)	
I Suffer from Limitations in My Everyday Life and Am Not Able to Perform My Usual Activities.	24 (29.6%)	1 (2.4%)	
Do Not Know	6 (3.6%)	1 (0.6%)	

**Table 3:** Self-Reported Long COVID Symptoms by Gender and Hospitalization Status.

	Hospitalized (n = 164)		*p* Value χ^2^	Non-Hospitalized (n = 164)		*p* Value χ^2^
Item	Male (n = 92)	Female (n = 72)		Male (n = 91)	Female (n = 73)	
Age Group (Years)			0.42			0.85
18–55	40 (43.4%)	26 (36.1%)		72 (79.1%)	58 (79.5%)	
56–Greater than 75	52 (56.5%)	46 (63.9%)		19 (20.9%)	15 (20.5%)	
Are you having new or lasting/ongoing symptoms (e.g., physical, cognitive, emotional, etc.) since you first tested positive for COVID-19?						
Yes	42 (45.6%)	39 (54.2%)	0.25	18 (19.7%)	23 (31.0%)	0.10
No	50 (54.4%)	33 (45.8%)		73 (80.3%)	50 (69.0%)	
How long have you been experiencing these symptoms?			0.25			0.24
<6 Months	8 (19.0%)	6 (15.5%)		8 (44.5%)	9 (39.1%)	
>6 Months	33 (78.6%)	33 (84.6%)		10 (55.6%)	14 (60.9%)	
Do Not Know	1 (2.4%)	-		-	-	
Since your experience of post-COVID, have you been able to return to your previous employment status?			0.02			0.91
Yes	43 (46.7%)	21 (29.1%)		66 (72.5%)	48 (65.7%)	
Partially	10 (10.9%)	5 (6.9%)		0 (0.0%)	6 (8.2%)	
No	11 (12.0%)	9 (12.5%)		5 (5.5%)	1 (1.4%)	
Other	3 (3.3%)	1 (1.4%)		1 (1.0%)	4 (5.4%)	
Missing	25 (27.2%)	36 (50.0%)		19 (20.9%)	14 (19.2%)	
Please select the choice that best describes how your symptoms could have had an impact on your usual, everyday activities.			0.13			0.49
My Usual, Everyday Activities are Not Impacted by My Symptoms.	5 (11.9%)	5 (12.8%)		12 (66.7%)	4 (17.4%)	
I Can Perform Most of my Usual Activities.	3 (7.1%)	5 (12.8%)		2 (11.1%)	6 (26.1%)	
I Sometimes Need to Stop or Cut Down on My Usual Activities.	9 (21.4%)	14 (35.9%)		2 (11.1%)	10 (43.4%)	
I Often Need to Stop or Cut Down My Usual Activities.	7 (16.7%)	3 (7.7%)		2 (11.1%)	1 (4.3%)	
I Suffer from Limitations in My Everyday Life and Am Not Able to Perform My Usual Activities.	13 (31.0%)			0 (0%)	1 (4.3%)	
Do Not Know	5 (11.9%)			-	1 (4.3%)	

2 participants refused to identify a gender.

**Table 4:** Self-Reported Long COVID Symptoms by Geographical Region.

	Hospitalized (n = 165)		*p* Value χ^2^	Non-Hospitalized (n = 165)		*p* Value χ^2^
Item	Metropolitan-Urban (n = 99)	Regional-Urban/Rural (n = 66)		Metropolitan-Urban (n = 99)	Regional-Urban/Rural (n = 66)	
Age Group (Years)			0.90			0.04
18–55	39 (39.4%)	26 (39.4%)		83 (83.8%)	46 (69.7%)	
56 –>75	60 (60.6%)	40 (60.6%)		16 (16.2%)	19 (28.8%)	
Refused	-	-		-	1 (1.5%)	
Gender (% Female)	40 (40.4%)	30 (45.5%)	0.48	46 (46.5%)	27 (40.9%)	0.34
Are you having new or lasting/ongoing symptoms (e.g., physical, cognitive, emotional, etc.) since you first tested positive for COVID-19?			0.25			0.23
Yes	45 (45.4%)	36 (54.5%)		25 (25.2%)	18 (27.3%)	
No	54 (54.5%)	30 (45.4%)		74 (74.7%)	48 (72.7%)	

## Data Availability

Data available on request due to restrictions, e.g., privacy or ethical. The data presented in this study are available on request from the corresponding author. The data are not publicly available due to participant confidentiality.

## Supplementary Materials

The following supporting information can be downloaded at: https://www.mdpi.com/article/10.3390/healthcare11091309/s1, Table S1: Checklist for Reporting of Survey Studies; File S2: Accessing Care and Services After COVID-19: A Patient Experience Survey.
