# Supplementary material for: Understanding the Experience of Long COVID Symptoms in Hospitalized and Non-Hospitalized Individuals: A Random, Cross-Sectional Survey Study
Source: Healthcare (Basel). 2023 May 3;11(9):1309. doi: 10.3390/healthcare11091309 (PMC10178853; doi:10.3390/healthcare11091309)
Supplement: Supplementary file 1 [file healthcare-11-01309-s001.zip › healthcare-2327769-supplementary.pdf]

Table S1: Checklist for Reporting of Survey Studies

Section/Topic	Item	Item Description	Reported on Page #
Title and Abstract			
	1a	State the word "survey" along with a commonly used term in title or abstract to introduce the study's design.	1
	1b	Provide an informative summary in the abstract, covering background, objectives, methods, findings/results, interpretation/discussion, and conclusions.	1
Introduction			
	2	Provide a background about the rationale of study, what has been previously done, and why this survey is needed.	1-2
	3	Identify specific purposes, aims, goals, or objectives of the study.	2
Methods			
Study Design	4	Specify the study design in the methods section with a commonly used term (e.g., cross-sectional or longitudinal).	2-3
	5a	Describe the questionnaire (e.g., number of sections, number of questions, number and names of instruments used).	3-4
	5b	Describe all questionnaire instruments that were used in the survey to measure particular concepts. Report target population, reported validity and reliability information, scoring/classification procedure, and reference links (if any).	3-4
Data Collection Methods	5c	Provide information on pretesting of the questionnaire, if performed (in the article or in an online supplement). Report the method of pretesting, number of times questionnaire was pre-tested, number and demographics of participants used for pretesting, and the level of similarity of demographics between pre-testing participants and sample population.	3
	5d	Questionnaire if possible, should be fully provided (in the article, or as appendices or as an online supplement).	3
	6a	Describe the study population (i.e., background, locations, eligibility criteria for participant inclusion in survey, exclusion criteria).	4-5
Sample Characteristics	6b	Describe the sampling techniques used (e.g., single stage or multistage sampling, simple random sampling, stratified sampling, cluster	4

		sampling, convenience sampling). Specify the locations of sample participants whenever clustered sampling was applied.	
	**6c**	Provide information on sample size, along with details of sample size calculation.	**3-4**
	**6d**	Describe how representative the sample is of the study population (or target population if possible), particularly for population-based surveys.	**3-4**
	**7a**	Provide information on modes of questionnaire administration, including the type and number of contacts, the location where the survey was conducted (e.g., outpatient room or by use of online tools, such as SurveyMonkey).	**4**
**Survey Administration**	**7b**	Provide information of survey's time frame, such as periods of recruitment, exposure, and follow-up days.	**4**
	**7c**	Provide information on the entry process:<br>–>For non-web-based surveys, provide approaches to minimize human error in data entry.<br>–>For web-based surveys, provide approaches to prevent "multiple participation" of participants.	**4**
**Study Preparation**	**8**	Describe any preparation process before conducting the survey (e.g., interviewers' training process, advertising the survey).	**3-4**
**Ethical Considerations**	**9a**	Provide information on ethical approval for the survey if obtained, including informed consent, institutional review board [IRB] approval, Helsinki declaration, and good clinical practice [GCP] declaration (as appropriate).	**3**
	**9b**	Provide information about survey anonymity and confidentiality and describe what mechanisms were used to protect unauthorized access.	**4**
**Statistical Analysis**	**10a**	Describe statistical methods and analytical approach. Report the statistical software that was used for data analysis.	**4**
	**10b**	Report any modification of variables used in the analysis, along with reference (if available).	**N/A**
	**10c**	Report details about how missing data was handled. Include rate of missing items, missing data mechanism (i.e., missing completely at random [MCAR], missing at random [MAR] or	**4**

		missing not at random [MNAR]) and methods used to deal with missing data (e.g., multiple imputation).	
	**10d**	State how non-response error was addressed.	**4**
	**10e**	For longitudinal surveys, state how loss to follow-up was addressed.	**N/A**
	**10f**	Indicate whether any methods such as weighting of items or propensity scores have been used to adjust for non-representativeness of the sample.	**N/A**
	**10g**	Describe any sensitivity analysis conducted.	**N/A**
**Results**	**11**	Report numbers of individuals at each stage of the study. Consider using a flow diagram, if possible.	**5**
**Respondent Characteristics**	**11b**	Provide reasons for non-participation at each stage, if possible.	**4-5**
	**11c**	Report response rate, present the definition of response rate or the formula used to calculate response rate.	**4**
	**11d**	Provide information to define how unique visitors are determined. Report number of unique visitors along with relevant proportions (e.g., view proportion, participation proportion, completion proportion).	**4**
**Descriptive Results**	**12**	Provide characteristics of study participants, as well as information on potential confounders and assessed outcomes.	**4-5**
	**13a**	Give unadjusted estimates and, if applicable, confounder-adjusted estimates along with 95% confidence intervals and p-values.	**5-10**
**Main Findings**	**13b**	For multivariable analysis, provide information on the model building process, model fit statistics, and model assumptions (as appropriate).	**N/A**
	**13c**	Provide details about any sensitivity analysis performed. If there are considerable amount of missing data, report sensitivity analyses comparing the results of complete cases with that of the imputed dataset (if possible).	**N/A**
**Discussion**			
**Limitations**	**14**	Discuss the limitations of the study, considering sources of potential biases and imprecisions, such as non-representativeness of sample, study design, important uncontrolled confounders.	**12**
**Interpretations**	**15**	Give a cautious overall interpretation of results, based on potential biases and	**11-13**

		imprecisions and suggest areas for future research.	
**Generalizability**	**16**	Discuss the external validity of the results.	**11-13**
**Other Sections**			
**Role of Funding Source**	**17**	State whether any funding organization has had any roles in the survey's design, implementation, and analysis.	**13**
**Conflict of Interest**	**18**	Declare any potential conflict of interest.	**13**
**Acknowledgements**	**19**	Provide names of organizations/persons that are acknowledged along with their contribution to the research.	**13**

File S2: Accessing Care and Services After COVID-19: A Patient Experience Survey

THE FIRST FEW QUESTIONS ARE ABOUT YOUR EXPERIENCE WITH COVID:

1. What month in 2021 did you test positive for COVID-19?
   - ❑ March
   - ❑ April
   - ❑ May
   - ❑ June
   - ❑ July
   - ❑ August
   - ❑ September
   - ❑ October
   - ❑ DON'T READ: Don't Know [continue to Q2]
   - ❑ DON'T READ: Refused [continue to Q2]

2. Were you ever admitted to the hospital for your initial COVID-19 infection or for your post-COVID symptoms?
   - ❑ Yes [continue to Q3]
   - ❑ No [continue to Q4]

3. What month in 2021 were you hospitalized for COVID-19? [Read out list of months if needed]
   - ❑ March
   - ❑ April
   - ❑ May
   - ❑ June
   - ❑ July
   - ❑ August
   - ❑ September
   - ❑ October
   - ❑ DON'T READ: Don't know [continue to Q4]
   - ❑ DON'T READ: Refused [continue to Q4]

4. Are you having new or lasting/ongoing symptoms (e.g. physical, cognitive, emotional, etc.) since you were treated in hospital for COVID-19?
   - ❑ Yes (Two questions a and b follow)
   - ❑ No [go to Q5]
   - ❑ DON'T READ: Don't know [go to Q5]
   - ❑ DON'T READ: Refused [go to Q5]

a. *If yes*, I am going to read 5 statements that describe how your symptoms could have had an impact on your usual everyday activities. Please select the one that best applies to you.
   - ❑ My usual, everyday activities are not impacted by my symptoms (i.e. I can perform all my usual activities).
   - ❑ I can perform most of my usual activities.
   - ❑ I sometimes need to stop or cut down on my usual activities.

- ❑ I often need to stop or cut down my usual activities.
- ❑ I suffer from limitations in my everyday life and am not able to perform my usual activities.
- ❑ DON'T READ: Don't know [go to Q5]
- ❑ DON'T READ: Refused [go to Q5]

b. *If yes to the previous question,* how long have you been experiencing these symptoms? *[Open response - participant provides a numeric answer in days, weeks, or months]*

- ❑ Less than one month
- ❑ 1-3 months
- ❑ 3-6 months
- ❑ More than 6 months
- ❑ DON'T READ: Don't know [continue to Q5]
- ❑ DON'T READ: Refused [continue to Q5]

THE NEXT FEW QUESTIONS ARE ABOUT INFORMATION FOR RECOVERY FROM COVID-19

Please answer 'yes' or 'no to following question:

5. Did you need any information about recovering from COVID-19?
   - ❑ Yes [continue with Q8]
   - ❑ No, did not need any information [skip to Q8]
   - ❑ DON'T READ: Don't know [continue to Q8]
   - ❑ DON'T READ: Refused [continue to Q8]

If person answers 'yes' to previous question 5:

Please answer with 'yes' or 'no to following question:

6. Did a healthcare provider give you any information about recovering from COVID-19?
   - ❑ Yes [continue with question a]
   - ❑ No [skip to question b]
   - ❑ DON'T READ: Don't know [skip to question 8]
   - ❑ DON'T READ: Refused [skip to question 8]

If person answers yes to Q6:

7. Which healthcare provider(s) gave you information about managing your recovery from COVID-19? Select all that apply. [Read list]
   - ❑ Family doctor
   - ❑ Specialist care provider at the hospital
   - ❑ Specialist care provider in an outpatient setting
   - ❑ Nurse

- ❑ Other healthcare providers (e.g., pharmacist, physiotherapist, dentist)
- ❑ DON'T READ: Don't know [go to Q9]
- ❑ DON'T READ: Refused [go to Q9]

b. ***If person answers no to Q6:***

8. Did you have to search for information on your own?
   - ❑ Yes [go to c]
   - ❑ No [go to Q9]
   - ❑ DON'T READ: Don't know [go to Q9]
   - ❑ DON'T READ: Refused [go to Q9]

c. If yes to this last question in b: Where did you find information about managing your recovery from COVID-19? Select all that apply.

- ❑ Government of Alberta website
- ❑ Alberta Health Services website
- ❑ Social media (Twitter, Facebook, Instagram, Tiktok)
- ❑ Other websites or media (blogs, podcasts, radio, medial websites, etc.)
- ❑ Support groups
- ❑ Other [open response]
- ❑ DON'T READ: Don't know [continue to Q9]
- ❑ DON'T READ: Refused [continue to Q9]

9. Please tell me how much you agree or disagree with the following statements. Most of the time, if you needed to get information to help you with your recovery following COVID-19, you were able to find or get it.
   - ❑ Strongly Agree
   - ❑ Agree
   - ❑ Not sure
   - ❑ Disagree
   - ❑ Strongly Disagree
   - ❑ DO NOT READ - Not applicable
   - ❑ DO NOT READ – Refused

10. Your chosen family and friends had the information they needed to support you in your recovery following COVID-19.
    - ❑ Strongly Agree
    - ❑ Agree
    - ❑ Not sure
    - ❑ Disagree
    - ❑ Strongly Disagree
    - ❑ DO NOT READ - Not applicable
    - ❑ DO NOT READ – Refused

I AM GOING TO ASK SOME QUESTIONS ABOUT USING? THE HEALTH SYSTEM TO HELP WITH YOUR RECOVERY

11. ***Please answer 'yes' or 'no' to the following questions:***
   - ❑ After leaving the hospital, did you see your family doctor because of new and/or lasting symptoms during your recovery from COVID-19?
   - ❑ Yes [go to questions a and b]
   - ❑ No [go to question c]
   - ❑ DON'T READ: Don't know [go to question c]
   - ❑ DON'T READ: Refused [go to question c]

a. *If yes to Q11.* Did you feel you had timely access to your family doctor to help you manage your new and/or lasting symptoms?
- ❑ Yes
- ❑ No
- ❑ DON'T READ: Don't know [continue with b]
- ❑ DON'T READ: Refused [continue with b]

b. If yes to Q11. Did your family doctor help you manage your new and/or lasting symptoms?

- ❑ Yes
- ❑ No
- ❑ DON'T READ: Don't know [go to Q12]
- ❑ DON'T READ: Refused [go to Q12]

c. If no to Q11. Did you see another healthcare provider?

- ❑ Yes
- ❑ No
- ❑ DON'T READ: Don't know [continue to Q12]
- ❑ DON'T READ: Refused [continue to Q12]

12. Were you referred to specialty healthcare providers to help manage your new and/or lasting symptoms? (e.g., referrals to physiotherapist, psychologist, specialist clinic, COVID-19 clinic, etc.)?
   - ❑ Yes [questions a, b and c follow}
   - ❑ No [go to Q13]
   - ❑ DON'T READ: Don't know [go to Q13]
   - ❑ DON'T READ: Refused [go to Q13]

a. *If answered yes to Q12,* which of these specialty healthcare provider(s) were you referred to? Select all that apply. [Read list]
- ❑ Physiotherapist
- ❑ Occupational therapist
- ❑ Respiratory therapist
- ❑ Mental health professional
- ❑ Social workers

- ❑ Specialist clinic
- ❑ COVID-19 clinic specialist
- ❑ Other
- ❑ DON'T READ: Don't know [continue to b]
- ❑ DON'T READ: Refused [go to Q13]

b. *If yes to Q12,* was your access to a referred specialty healthcare provider(s) timely (i.e. no major delays to get in to see care provider)? [Please answer with yes or no]

- ❑ Yes
- ❑ No
- ❑ DON'T READ: Don't know [continue to c]
- ❑ DON'T READ: Refused [continue to c]

c. *If yes to Q12,* did the referral/referrals help you with your recovery following COVID-19?

- ❑ Yes
- ❑ No
- ❑ Somewhat
- ❑ DON'T READ: Don't know [continue to Q13]
- ❑ DON'T READ: Refused [continue to Q13]

13. Did you access any other care services that did not need a referral? (e.g., physiotherapy, mental health, massage therapy, acupuncture, chiropractor, naturopaths)

- ❑ Yes [go to question a that follows]
- ❑ No [go to question 14]
- ❑ DON'T READ: Don't know [go to Q14]
- ❑ DON'T READ: Refused [go to Q14]

a. *If answered yes to Q13,* what care services did you access without a referral? Select all that apply. [Read the list].

- ❑ Physiotherapy
- ❑ Mental health
- ❑ Massage therapy
- ❑ Acupuncture
- ❑ Chiropractor
- ❑ Naturopathy
- ❑ Other [open response]
- ❑ DON'T READ: Don't know [continue to Q14]
- ❑ DON'T READ: Refused [continue to Q14]

14. During any time after you left the hospital, did you need to visit the emergency room or urgent care while waiting to see a healthcare provider for your new or lasting symptoms from COVID-19?

- ❑ Yes
- ❑ No

- ❑ DON’T READ: Don’t know
- ❑ DON’T READ: Refused

15. Did you have full or partial private insurance coverage for any of the referred or non-referred services you accessed?
    - ❑ Yes
    - ❑ No
    - ❑ DON’T READ: Don’t know
    - ❑ DON’T READ: Refused

16. Did you have to pay or partially pay out-of-pocket for some or any of the services you accessed?
    - ❑ Yes [question a follows]
    - ❑ No [go to question 17]
    - ❑ DON’T READ: Don’t know [go to Q17]
    - ❑ DON’T READ: Refused [go to Q17]

    a. *If yes to previous Q16 w*hat services did you have to pay or partially pay for? [open text]
    - ❑ DO NOT READ – Don’t know [continue to Q17]
    - ❑ DO NOT READ – Refused [continue to Q17]

Please tell me how much you agree or disagree with the following statements:

17. If you needed care and supports to help you with your recovery from COVID-19, you were able to locate and access them.
    - ❑ Strongly Agree
    - ❑ Agree
    - ❑ Not sure
    - ❑ Disagree
    - ❑ Strongly Disagree
    - ❑ DO NOT READ - Don’t know [continue to Q18]
    - ❑ DO NOT READ – Refused [continue to Q18]

18. Most of the time, you were able to get the care you needed in a timely manner.
    - ❑ Strongly Agree
    - ❑ Agree
    - ❑ Not sure
    - ❑ Disagree
    - ❑ Strongly Disagree
    - ❑ DO NOT READ - Don’t know [continue to Q19]
    - ❑ DO NOT READ – Refused [continue to Q19]

19. The healthcare providers you saw were usually informed and demonstrated confidence about managing your recovery from COVID-19.
    - ❑ Strongly Agree
    - ❑ Agree

- ❑ Not sure
- ❑ Disagree
- ❑ Strongly Disagree
- ❑ DO NOT READ - Don't know [continue to Q20]
- ❑ DO NOT READ – Refused [continue to Q20]

20. The healthcare providers you visited usually knew where to refer you for other recovery supports.
    - ❑ Strongly Agree
    - ❑ Agree
    - ❑ Not sure
    - ❑ Disagree
    - ❑ Strongly Disagree
    - ❑ DO NOT READ - Don't know [continue to Q21]
    - ❑ DO NOT READ – Refused [continue to Q21]

21. Most of the time, the referral(s) your healthcare provider gave you were a good choice for helping you and managing your recovery from COVID-19.
    - ❑ Strongly Agree
    - ❑ Agree
    - ❑ Not sure
    - ❑ Disagree
    - ❑ Strongly Disagree
    - ❑ DO NOT READ - Don't know [continue to Q22]
    - ❑ DO NOT READ – Refused [continue to Q22]

THE NEXT FEW QUESTIONS ARE ABOUT YOU. THIS INFORMATION WILL BE USED TO GROUP OUR RESULTS

22. What is your age group?
    - ❑ 18-24 years
    - ❑ 25-40 years
    - ❑ 41-55 years
    - ❑ 56-65 years
    - ❑ 66-75 years
    - ❑ >75 years
    - ❑ DON'T READ: Refused [continue to Q23]

23. How do you identify your gender? [Read out list]
    - ❑ Woman/Female
    - ❑ Man/Male
    - ❑ Two-spirit
    - ❑ Non-binary/Genderfluid
    - ❑ Other – *open comment*
    - ❑ DON'T READ: Refused [continue to Q24]

24. Which city or town do you currently live in, or near? [open text]
    - ❑ DON'T READ: Refused [continue to Q25]

25. Which of the following best describes your ethnicity? Please select

all that apply to you.

- ❑ Arab
- ❑ Black
- ❑ Caucasian
- ❑ Chinese
- ❑ Filipina
- ❑ Indigenous
- ❑ Japanese
- ❑ Korean
- ❑ Latin American
- ❑ South Asian (e.g., Pakistani, East Indian)
- ❑ Southeast Asian (e.g., Vietnamese, Thai)
- ❑ West Asian (e.g., Iranian, Afghan)
- ❑ Other [open text]
- ❑ DON'T READ Refused [continue to Q26]

26. What was your employment status before you got COVID-19?

- ❑ Full-time
- ❑ Part-time
- ❑ Casual
- ❑ Student
- ❑ Not employed
- ❑ Retired
- ❑ Other [open response]
- ❑ DON'T READ: Don't know [continue to Q27]
- ❑ DON'T READ: Refused [continue to Q27]

27. Following your recovery from COVID-19, have you been able to return to your previous employment status?

- ❑ Yes
- ❑ Partially (modified hours)
- ❑ No
- ❑ Other [open response]
- ❑ DON'T READ: Don't know [continue to Q28]
- ❑ DON'T READ: Refused [continue to Q28]

28. ***Finally:*** Is there anything else about your recovery experiences you wish to share? [open text]

- ❑ NO Response
